# Selecting the appropriate hurdles and endpoints for pentilludin, a novel antiaddiction pharmacotherapeutic targeting the receptor type protein tyrosine phosphatase D

**DOI:** 10.3389/fpsyt.2023.1031283

**Published:** 2023-04-17

**Authors:** George R. Uhl

**Affiliations:** ^1^Departments of Neurology and Pharmacology, University of Maryland School of Medicine, Neurology Service, VA Maryland Healthcare System, Baltimore, MD, United States; ^2^Department of Mental Health, Johns Hopkins Bloomberg School of Public Health, Baltimore, MD, United States

**Keywords:** receptor type protein tyrosine phosphatase, lapse doses, relapse, antiaddiction drug development, inhibitors of protein tyrosine phosphatase

## Abstract

Substance use disorders provide challenges for development of effective medications. Use of abused substances is likely initiated, sustained and “quit” by complex brain and pharmacological mechanisms that have both genetic and environmental determinants. Medical utilities of prescribed stimulants and opioids provide complex challenges for prevention: how can we minimize their contribution to substance use disorders while retaining medical benefits for pain, restless leg syndrome, attention deficit hyperactivity disorder, narcolepsy and other indications. Data required to support assessments of reduced abuse liability and resulting regulatory scheduling differs from information required to support licensing of novel prophylactic or therapeutic anti-addiction medications, adding further complexity and challenges. I describe some of these challenges in the context of our current efforts to develop pentilludin as a novel anti-addiction therapeutic for a target that is strongly supported by human and mouse genetic and pharmacologic studies, the receptor type protein tyrosine phosphatase D (PTPRD).

## Introduction

### Urgent public health needs

Development of safe and effective medications to aid prevention and treatment of stimulant, opioid and stimulant + opioid use disorders are urgent public health needs. I can describe some of the statistics using published materials and some *via* links to websites for material that is not published.

### Stimulants

Almost 20 metric tons of amphetamines and almost 9 metric tons of lisdexamphetamine are prescribed *via* >30 million annual prescriptions in the US (1). Many were prescribed chronically for indications including ADHD and narcolepsy.^1^ 1.8% of the US population reported missuse of a prescribed stimulant.^2^ 0.7% of the US population used an amphetamine from a licit or illicit source, almost 7.5 million Americans reported cocaine or methamphetamine use and almost 1.8 million reported a cocaine or amphetamine use disorder (see text footnote 2). Despite the lack of any FDA-approved medication for cocaine or methamphetamine use disorders, there are about 200,000 annual admissions to a stimulant use disorder treatment program (see text footnote 2).

### Opioids

More than 142 million opioid prescriptions are written annually in the US,^3^ many prescribed chronically for chronic pain. More than 10 million Americans misuse opioids/year (745,000 using heroin and 9.7M prescription pain relievers) and almost 50,000/year die from this use.^4^

### Stimulants + opioids

There is increasing co use of opioids along with stimulants (though not necessarily from “speedball” preparations that are intentionally co-injected). Past-year methamphetamine use among people using heroin rose from 23 to 37% between 2015 and 2018, while past year of methamphetamine use among people using prescription opioids increased from 5 to 8% over this period (2). Among people reporting past-month heroin use, past-month methamphetamine use increased from 9 to 30% between 2015 and 2017 (3). Fifty Percent of a 2015 sample of people who inject drugs reported injecting both heroin and methamphetamine (4).

Drug overdoses, both fatal and non-fatal, now include increasing numbers of individuals who use both opioids and stimulants (5, 6). Opioid users experience adverse features that are even more prevalent in opioid + stimulant co-users. Co-users have higher prevalence of injection drug use, serious mental illness, hepatitis B or C (7) emergency department visits, days hospitalized, utilization of social services, involvement with the criminal justice system (8) as well as overdose (4, 6).

### Treatment

Such consequences are among the reasons that many individuals with opioid or opioid + stimulant use disorders attempt to quit. More than 750,000, 560,000, 490,000 and 118,000 Americans seek treatment for disorders of use of heroin, amphetamines, cocaine and “stimulants” annually, respectively.^5^ Agonist-like, antagonist and other therapeutics for opioid use disorders provide benefits but remain suboptimal for many for reasons that include variable adherence to these regimens (9, 10). More than 300,000 and 175,000 Americans receive methadone or buprenorphine annually, respectively;^6^ about 20,000 receive naltrexone.

Despite available treatments, relapses (especially early in treatment) are frequent. Relapse occurred within a week in 59% of a group of individuals seeking to quit opioid use (11) and within a month in 37% of a group of individuals seeking to quit methamphetamine use (12). Rates of relapse decline during subsequent periods.

Current FDA approved agonist and antagonist therapies for opioid use disorder remain suboptimal for many. There are no FDA-approved medications for preventing disorders of use of opioids or stimulants, none for treating stimulant abuse disorders and none approved for disorders of combined opioid + stimulant use.

## Prevention and treatment

### Prevention

Pharmacologic strategies to *prevent* development of substance use disorders in those who are prescribed opioids or stimulants could reasonably focus on goals that include reducing the abuse liability of these substances while maintaining their therapeutic benefits for indications that include reductions in pain, symptoms of restless leg syndrome (RLS), symptoms of attention deficit-hyperactivity disorder (ADHD) and the daytime sleepiness of narcolepsy.

### Animal model data

Tests in experimental animals and humans can evaluate abuse liability with sufficient reliability that they are used to place new substances in the appropriate regulatory schedule (13, 14). There are good animal models for several types of pain that are often validated in humans (15). Although animal models for ADHD or RLS are perhaps not as well validated (16, 17), there is still reasonably good ability to identify compounds that reduce abuse liability of opioid analgesics and stimulants while preserving many likely therapeutic benefits. Such identification in animal models can lead to assessments of such selectivity in human laboratory studies that test aspects of human abuse liability (14). Taken together, human and animal data that indicate lower abuse liability might support less restrictive scheduling of combined stimulant + agent that reduces abuse liability and/or opioid + agent that reduces abuse liability. Below, I consider some of the hurdles that a candidate antiaddiction therapeutic, pentilludin, might need to clear to be used in these contexts. Our discussion overlaps with recent work considering different “endpoints” for antiaddiction therapeutics (18–23) but approaches this topic in a different way (e.g., from the perspective of a novel agent's development).

### Treatment

Pharmacologic strategies for treatment include facilitating initial abstinence, often in the face of withdrawal symptoms. Sustaining abstinence often requires success in the face of pharmacologic and behavioral effects of “lapse” doses of an abused substance as well as behavioral effects of exposures to stimuli that have been strongly associated with prior drug experiences (24). Current animal models cover some of these features (25). Studies of behavioral components of reinstatement triggered by experimenter-administered drug doses, pain or stress provide significant data (26–28). Below, I consider the hurdles that pentilludin would need to clear for use in reducing the reinstatement-promoting effects of lapse doses of stimulants or opioids and contrast this with hurdles required for less-targeted use in other aspects required to achieve and sustain abstinence.

A number of recent publications note interest in using reductions in use and/or quantity frequency as an indication for treatments for stimulant use disorders (18–22). I thus also consider the hurdles that pentilludin would need to clear should similar consensus develop *re* endpoints of reduced use in individuals with disorders of use of stimulants, opioids or stimulants + opioids.

## PTPRD and pentilludin

### PTPRD's phosphatase as a target for novel antiaddiction therapeutics

PTPRD, the receptor type protein tyrosine phosphatase D, is now a strongly-supported target for antiaddiction medication based on human, mouse model and *in vitro* data. Human genetic results (29) associate common variation in PTPRD with vulnerability to develop a substance use disorder [e.g., polysubstance (30–32), opioid (33), alcohol (34)]. PTPRD variation is also associated with the abilities to quit (smoking (35, 36), use of opioids (37) and alcohol when aided by naltrexone [though not acamprosate (38)]. There are PTPRD associations with individual differences in a specific constellation of rewarding responses to amphetamine administration (39).

**Figure 1:**
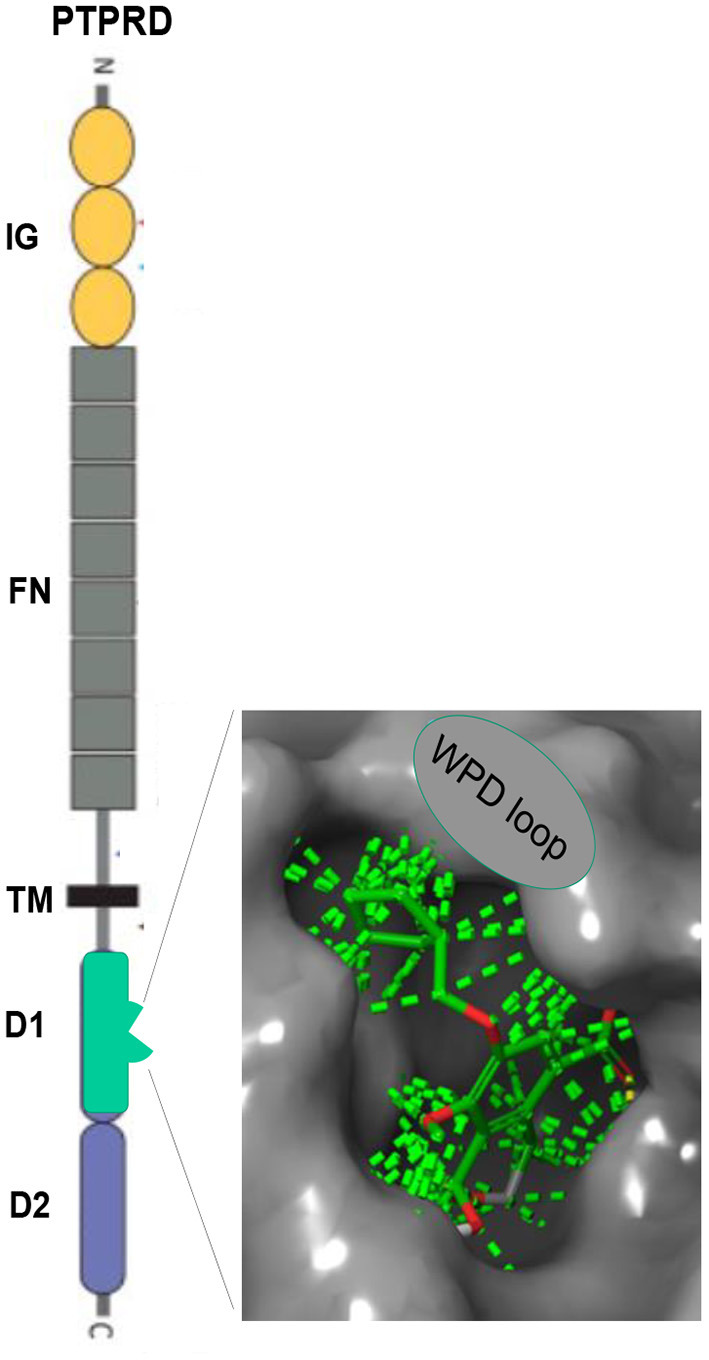
PTPRD. Extracellular immunoglobulin (IG) and fibronectin (FN) domains, transmembrane (TM) domain, D1 intracellular phosphatase domain. *Enlargement:* Pentilludin (NHB1109) bound to PTPRD phosphatase catalytic site and WPD loop. Dashed lines: van der Walls interactions.

PTPRD is a highly-expressed, largely-neuronal, substantially synaptic, single transmembrane protein (Figure 1) that (likely) transduces signals from binding to extracellular ligands (40) to alter activity of its intracellular phosphatase (41). Synaptosomal proteomic, *in situ* hybridization, single cell RNAseq and electron and light microscopic immuohistochemical data support these conclusions *re* localization (42–44).^7^ Reported PTPRD extracellular binding partners include slit/trk, interleukin-1 receptor like and accessory proteins, synaptic adhesion-like molecules (SALMs) and the peptide asprosin (45–50). Substrates for PTPRD's phosphatase include proteins that regulate synaptic strength and maturation (51). Processes of PTPRD-expressing neurons grow when their PTPRD makes homomeric bonds with PTPRD expressed by adjacent cells (52). Cerebral cortical, ventral midbrain, striatal/ accumbens, reticular thalamic and other circuits that express PTPRD mRNA in likely glutamatergic, GABAergic, cholinergic and dopaminergic neurons are likely to develop and adapt differently when they express PTPRD at differing levels (53).

### Results from mouse models of common human allelic PTPRD variation

We have identified robust, 60- 70% individual differences in brain levels of expression of PTPRD mRNA (54) from human subjects with major vs. minor PTPRD SNP alleles. By contrast, PTPRD lacks common missense variants (53).

PTPRD knockout mice with only one wildtype gene copy and 50% constitutive alterations in levels of PTPRD expression (e.g., heterozygotes) thus model effects of common human PTPRD allelic variation. These mice are similar to wildtype littermates in tests of nociception (hotplate, tailflick), memory (Morris water maze), fear/anxiety (dark box emergence, thigmotaxis) and motor abilities (screen hang time, locomotion, rotarod) (54).

Mice with reduced PTPRD expression display sizable reductions in stimulant reward as assessed by conditioned place preference (CPP, 10 mg/kg cocaine) or self-administration (55).

### Results from synthesis and testing of 7-BIA and 70 novel analogs identification of pentilludin (NHB1109)

Despite concerns that phosphatases were “undruggable” (56), we reported a lead compound PTPRD phosphatase inhibitor, 7-BIA, in 2018 (55). We followed this discovery with further structure-activity work testing more than 70 analogs. We identified 10 congeners with greater potency than 7-BIA and several that are more selective (57).

**Figure 2:**
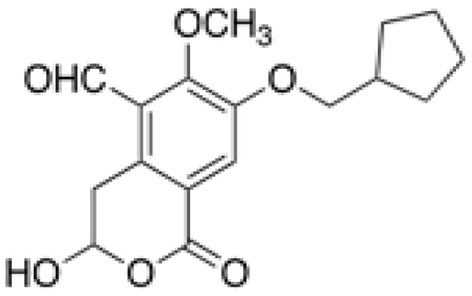
Pentilludin (NHB1109).

NHB1109, which I now name pentilludin, is a 7-position substituted cyclopentyl analog that, like 7-BIA, appears to provide pseudoirreversible inhibition of PTPRD's phosphatase (Figure 2) (57). It displays more potency and more selectivity vs. 7-BIA with respect to both close family members (PTPRS and PTPRF) and other phosphatases at which 7-BIA displays some potency, including PTPRJ and PTPN1/PTP1B. IC_50_ values are ≥10^−4^ M at 12 other receptor- and non-receptor type protein tyrosine phosphatases tested and ≥10^−5^ M in EUROFINS screen for targets of current drugs.

### Initial hurdles for pentilludin (NHB1109)

Pentilludin has cleared many initial hurdles in addition to the *in vitro* specificity noted above. Dose limiting toxicity comes from doses >10 x those that reduce cocaine reward. Mice treated daily with 200- 2,000 mg/kg gavage doses reduce food/water intake over serval days and lose weight (57). These results fit with recent observations that PTPRD's phosphatase serves as a receptor for the orexigenic actions of a “positive feedback” signal from fat cells, asprosin (50).

### Initial effects of pentilludin (NHB1109) pretreatments on stimulant and opioid reward

Pentilludin has replicated and extended the reward-reducing effects of acute PTPRD phosphatase inhibition displayed by our initial lead compound PTPRD phosphatase inhibitor 7-BIA (55).

The lack of deal-breaking toxicities and the presence of evidence for reduced stimulant reward suggest that pentilludin has cleared significant hurdles and encouraged us to move forward with its development as an antiaddiction compound.

## Evidence bearing on hurdles for development as an antiaddiction therapeutic

### Selecting the appropriate next hurdles for pentilludin

Development of novel small molecules for use in clinical addiction-related contexts requires substantial good laboratory practice (GLP) studies to seek evidence for toxicities investigational new drug application (IND), first doses in human research volunteers, repeated doses in humans, and dosing in humans along with addictive substance doses.

For purposes of this article, I focus on the ways in pentilludin or any other reward-reducing pharmacotherapeutic might be developed and deployed in light of the regulatory and other hurdles that these pathways place before its development. Another frame for this discussion: what useful endpoints might be most appropriately targeted by the actions that I believe PTPRD phosphatase inhibitors will provide? Prior to this discussion, I return to details of genetic and other evidence that might provide clues to which subsequent hurdles pentilludin might face with the most confidence, *a priori*.

### Some details from human evidence

Reevaluating details of selected human genetic studies supports testable hypotheses concerning the likely clinical influences of inhibitors that reduce the effects mediated by PTPRD. I evaluate these ideas in the context of the associations of common PTPRD haplotypes with substantial individual differences in levels of brain PTPRD expression (54).

### Stimulant reward

Hart et al. provided a clinical composite “factor 1” derived from responses of genotyped research volunteers as they experienced the effects of 10 mg oral amphetamine doses in a laboratory setting (39). Factor 1 came from sparse factor analyses of responses to items on the Profile of Mood States, Drug Effects Questionnaire and Addiction Research Center Inventory questionnaires. These authors generously confirmed to us that their 9p genomic association with factor 1 identified PTPRD. Pharmacologic reductions in PTPRD activity are thus likely to reduce “factor 1” effects of amphetamine in a) increasing friendliness, elation, vigor, feel high, want more, like, amphetamine-like, benzedrine-like, marijuana-like and morphine/benzedrine group-like responses and b) decreasing ratings of depression, fatigue, confusion and pentobarbitol-chlorpromazine-alcohol group sedation.

### Ability to quit use of addictive substances from different classes

The settings in which ability to reduce or quit substance use has been associated with variation in PTPRD may also provide clues. We identified polygenic association of PTPRD variation with biochemically-confirmed success in smoking cessation in participants in three clinical trials aided by nicotine replacement or bupropion (36), in a trial aided by denicotinized cigarettes (58), in a trial aided by precessation nicotine replacement (59) and in a nicotine replacement community trial (60). We also identified PTPRD associations in comparisons of former vs. current smokers (58). Cox et al. identified PTPRD associations in comparisons of individuals who displayed lifetime opioid dependence diagnoses and who (a) self-reported abstinence >1 year vs. (b) continued to use (e.g., any abstinence <6 mos) (37). Biernacka et al. evaluated data from studies of pharmacologic effects on alcohol abstinence and identified robust PTPRD associations with time to relapse and time to relapse to heavy drinking in analyses of data from naltrexone treated subjects, though not in analyses of subjects treated with acamprosate (38). Interestingly, naltrexone responsiveness has been associated with drinking associated with reward seeking, while acamprosate responsiveness has been associated with drinking to seek relief from negative affect (61). These alcohol results, combined with data from smoking cessation and ability to quit opioid use, are consistent with the idea that antiaddiction agents that reduce PTPRD activity should aid reduction in use and/or abstinence from addictive substances of several classes.

### Vulnerability to develop a substance use disorder of several classes

Vulnerability to develop a substance use disorder has been repeatedly associated with genomic variants at the PTPRD locus, beginning with our initial identification of a SNP in this chromosomal region using 10,000 SNP microarrays (30). We found PTPRD associations in comparisons of amphetamine dependent subjects to matched controls (62), research volunteers dependent on at least one illicit substance vs. corecruited or convenience controls (32, 63) and in more population- representative samples (64, 65). There are PTPRD copy number variant associations with vulnerability to develop opioid dependence (33). Agents that reduce PTPRD activity might thus be able to reduce development of dependence on addictive substances with which they were coadministered.

### Evidence against toxicities

There is also human evidence that speaks to the likelihood that pharmacologically-modified effects mediated by PTPRD would be oncogenic or provide irreversible toxicities. Several papers term PTPRD a “tumor suppressor” gene based on e.g., its abilities to alter cancer-related phosphorylation pathways (66). However, PTPRD variation has not been reproducibly associated with any common cancer in genetic association studies [bladder (67), breast (68), colon (69), endometrial (70), kidney (71), leukemia (72), liver (73), lung (74), melanoma (75), non-Hodgkin lymphomas [e.g., (76)], pancreatic (77), prostate (78) or thyroid (79)]. Mice with reduced PTPRD expression fail to develop tumors at ages up to 24 mos. Carcinogenicity risks of agents that alter PTPRD seem unlikely to be greater than those of agents that influence any novel target.

There is also evidence from accidental human ingestions of Jack o'lantern mushrooms (*Omphalotus illudens*) (80, 81). These mushrooms contain the illudalic acid compounds that have activities at PTPRD-related phosphatases and provide the core of pentilludin's structure (82). They also contain compounds with muscarinic cholinergic activities (83). There has been no lethality or persisting sequelae noted after > 60 reported cases of accidental ingestion of these mushrooms. The ingestions do produce nausea/emesis. These symptoms have been attributed to the mushrooms' muscarinic effects by authors including a physician who ingested them (81). It is also possible that pentilludin, especially at high doses, will act at PTPRD to produce anorexia/nausea in humans. PTPRD is expressed in the arcuate hypothalamic neurons that express the orexigenic agouti related peptide AgRP (50) as well as in human enteric neurons (84) and in brainstem sites of cholinergic inputs to the cervical ganglia that innervate the stomach and small intestine. We have identified reduced food intake in mice treated with single doses of pentilludin that are >10 times higher than those that reduce cocaine and opioid reward (57). Data from accidental human Ingestions of *Omphalotus illudens* do provide evidence for the lack of other idiosyncratic human responses to illudalic acid doses that is likely to be pertinent for the illudalic acid analog pentilludin.

### Human evidence limitations

The cumulative likelihood that all of the nominally-significant genetic associations cited above is due to chance is exceedingly small. When combined with the compelling mouse model and pharmacologic data, the *a posteriori* probabilities that PTPRD associations are due to chance are even lower. Nevertheless, none of these individual associations reproducibly meet the ultraconservative *p* < 10^−8^ Bonferroni corrected *p*-value required to declare “genome wide significance”. The PTPRD SNPs or copy number variants that provide these associations are not the same across all studies. All studies of addiction genetics do not identify PTPRD. The size of these human associations also provides a caution: the effects of common PTPRD genomic variation are likely to be modest when compared to the cumulative effects of other genetic and environmental variation on individual differences in vulnerability to develop a substance use disorder, reward from administration of addictive substances or ability to reduce use/abstain from use.

All in all, with the human genetic, mouse genetic and human mushroom experience, cited above we thus have substantial confidence that “on target” pentilludin actions at PTPRD are likely to be well tolerated at the proposed therapeutic doses, with an acceptable therapeutic index.

### Synthesis of human and animal evidence

The large effects on stimulant reward observed in our animal model studies (55), when we control other genetic and environmental features, suggest that both genetic and pharmacologic modulation of PTPRD activities can display robust effects. We thus seek a developmental pathway for the PTPRD phosphatase inhibitor pentilludin on which we can demonstrate clinical benefits in a setting in which these benefits will be less likely to be obscured by the effects of variation in other genetic and environmental influences.

Pragmatically, we wish to select the hurdles that pentilludin will be most likely to clear during further development with the support that is available to us. Another way to frame this: we need to select the most appropriate endpoint for pentilludin's initial use. We provide several possible examples below.

## Hurdles for development of antiaddiction therapeutic in several contexts

### Potentially-modest hurdle: Reducing reward from “lapse” doses of stimulants during the initial period of abstinence

Reward from “lapse” doses of stimulants is likely to contribute to the reasons why relapse is so frequent when individuals with stimulant use disorders attempt to quit. A relapse occurred within a month in 37% of a group of individuals seeking to quit methamphetamine use (12). Rates of relapse decline during subsequent periods. Interventions that reduce the reward from “lapse” doses of stimulants taken during the key 1st weeks of a quit attempt could thus reduce relapse rates and aid longer term abstinence. Quantitative tests can biochemically confirm abstinence, exposure to modest “lapse” doses or relapse in appropriately-collected urine samples (85, 86). In this context, endpoints could be both (1) number of positive urine tests and (2) number of strikingly positive tests indicative of relapse in individuals who have previously displayed a modest positive indicative of lapse dosing.

“Lapse” is perhaps best defined in the smoking field, where smoking a single experimenter-administered cigarette can more than double the risk of continued smoking within the next 24 h (87). Such human evidence is complemented by animal studies that document how robustly experimenter-administered “priming” doses increase subsequent relapse-like efforts to self-administer drugs during periods of “abstinence” (28). Reducing the reward from “lapse” stimulant doses taken during the 1st week of attempted stimulant abstinence should thus provide a significant benefit for individuals seeking to quit use of stimulants.

IND-enabling studies for development of pentilludin to reduce reward from “lapse” doses of stimulants during the initial period of abstinence could be relatively tractable. Several weeks' pentilludin dosing in two species could be coupled with simulated “lapse” doses of stimulants. Since there are likely fewer than 200,000 individuals treated with any pharmacological adjunct to aid abstinence from a stimulant use disorder^8^ in the US, use of pentilludin to aid abstinence by reducing the reward from lapse doses of stimulants sampled during the 1st week of abstinence might even provide an “orphan” indication. Achieving orphan designation could encourage commercial partnerships to continue development by providing tax benefits and a period of exclusivity.^9^

The benefits of tractable IND-enabling work, possible orphan indication, ability to biochemically confirm both lapse dosing and abstinence and a plausible link to the reward conferred by stimulants appears to raise only moderately-high hurdles to development of pentilludin for this indication. However, this indication would target a market of only modest size. We do not have a clear indication of the fraction of the total variance in relapse to stimulant use that comes from PTPRD-sensitive stimulant reward during lapse doses vs. the fraction that comes from other genetic and environmental variables. The hurdle provided by this indication for pentilludin may thus be higher than we anticipate, *a priori*.

### Potentially-higher hurdle: Reducing abuse liability from prescribed stimulants and/or opioids

Prevention of the substance use disorders that arise from use of prescribed drugs would provide a large clinical impact. This impact could come from strategies that reduced the reward that these prescribed drugs provide while maintaining their therapeutic benefits. A practical manifestation of this impact could be lower scheduling of combination products (e.g., pentilludin + stimulant or pentilludin + opioid).

Human laboratory and experimental animal assessments of reward have been key to Drug Enforcement Administration (DEA) assignment of appropriate regulatory schedules to new drugs (Box 1^10^), with consultation by FDA (13). Postmarketing data identifying frequencies of missuse and abuse in the community add valuable information (13). Possible endpoints in this developmental pathway would be demonstration of lower signs of abuse liability in standard testing paradigms and thus reduced scheduling for combination products.

Box 1 **Schedules for controlled substances.**

**Schedule I:** Drugs with no currently accepted medical use and a high potential for abuse. (ex: heroin, LSD, marijuana (cannabis), 3,4-methylenedioxy-methamphetamine (ecstasy), methaqualone, peyote.

**Schedule II:** Drugs with a high potential for abuse, potentially leading to severe psychological or physical dependence, that are considered dangerous. (ex: combination products with <15 mg hydrocodone per dosage, cocaine, methamphetamine, amphetamine, methadone, hydromorphone, meperidine, oxycodone, fentanyl, methylphenidate.

**Schedule III:** Drugs with a moderate to low potential for physical and psychological dependence, less than Schedule I/ II drugs but more than Schedule IV. (ex: products containing < 90 mg codeine per dosage, ketamine, anabolic steroids, testosterone.

**Schedule IV:** Drugs with a low potential for abuse and low risk of dependence. (ex: diazepam, tramadol, alprazolam, carisoprodol, propoxyphene, lorazepam, pentazocine, zolpidem).

**Schedule V:** Drugs with lower potential for abuse than Schedule IV, many containing limited quantities of certain narcotics and often used for antidiarrheal, antitussive, and analgesic purposes. (ex: cough preparations with <200 mg codeine/100 ml, diphenoxylate/atropine, difenoxin/atropine, pregabalin, attapulgite).

One set of hurdles for developing pentilludin for use in this context relates to the magnitude of its effects: could co-administration of pentilludin with a Schedule II stimulant or opioid reduce abuse liability sufficiently to allow the combination product to be marketed as Schedule III? It is fortunate that we do have genetic association of PTPRD variation with individual differences in rewarding responses to laboratory-administered amphetamine doses (*noted above*). Assessments using many of the same instruments has provided data that has been accepted by regulatory agencies to schedule new drugs in the past.^11^

Another set of hurdles results from the chronicity of treatment: can IND-enabling and other studies adequately reflect the years-long patterns of use of prescribed stimulants and, for some, opioids?

An additional hurdle come from the differences in pharmacodynamic properties of pentilludin (a pseudoirreversible agent with an apparent days-long physiological half-life) vs. those of e.g., amphetamine or oxycodone, with shorter physiological half-lives.

Producing a robust abuse-resistant combination formulation that deters extraction of the opioid or stimulant provides another hurdle.

And, finally, assuring that combination formulations retain human benefits in reducing pain, combatting ADHD or RLS symptoms or reducing daytime sleepiness of narcoleptics provides a significant hurdle as well.

Despite these hurdles, the potential for preventing development of substance use disorders by marketing pentilludin-containing stimulant and opioid combination products with reduced abuse liability and correspondingly-less restrictive scheduling remains a powerfully attractive idea. Such marketing would then have to clear a final hurdle: Real world post marketing surveillance data that demonstrated less abuse (13).

### Potentially-higher hurdle: Aiding initiation and maintenance of abstinence in individuals with ongoing stimulant or opioid use disorders

Established disorders of stimulant and opioid use are likely to be maintained by complex polygenic genetic and environmental factors.^12^ A pharmacologic “magic bullet” that could arrest such ongoing disorders without unacceptable side effects in all abusers would provide huge societal benefits and has therefore been sought. However, the complex interplay of habitual and learned behaviors with the pulls exerted by both pharmacological reward and reduction in withdrawal's aversive features provides a daunting hurdle for any antiaddiction pharmaceutic to clear.

One conceptual approach to this “magic bullet” thinking considers the different ways in which individuals with substance use disorders might sustain their use and thus come to treatment *via* different pathways. Studies of alcohol use disorders have sought to separate drinkers who largely drink for the reward that alcohol provides and those who largely drink to mitigate negative features of their lives and affects (88). As noted above, these studies have identified more prominent benefits of naltrexone for those who drink to experience reward and more benefits of acamprosate for those who drink to reduce negative affect and feelings. PTPRD variants provide strong association with ability to reduce alcohol use with naltrexone, without any evidence for a strong or even moderate-strength association with ability to reduce alcohol use with acamprosate (38). PTPRD effects could thus plausibly be more prominent in aiding reductions in or abstinence from stimulants or opioids in the subset of individuals whose use was the most strongly maintained by the reward that they obtain from these substances. Possible endpoints for this approach could include reduced or absent urinary levels of abused substances in unselected treatment-seeking substance users. Another endpoint could focus on the individuals whose substance use disorders had been most maintained by the rewarding effects of the substance.

Hurdles to development of pentilludin to aid initiation of and/or maintenance of reductions in or abstinence from use of addictive substances are thus daunting, but perhaps not impossible. In an ideal scenario, drug addiction investigators would identify the stimulant or opioid users whose use was most dependent on the reward that these drugs provide vs. habitual use or use to relieve negative features. In an even more ideal scenario, drug addiction investigators would also provide solid data (*re* benefits) that would lower barriers to licensing novel agents based on reductions in use of addictive drugs rather that complete abstinence. In such a setting, one of the highest hurdles to testing pentilludin to aid initiation of reduced use/abstinence in the most reward-dependent users might be studies identifying little pentilludin toxicity with co-use of the multiple addictive substance that are characteristic of many with substance use disorders. Aid in maintaining abstinence, once achieved, could be analogous to reducing reward from “lapse” doses, as noted above. Hurdles to aiding initiation and maintenance of abstinence in individuals with the most reward-dependent ongoing stimulant or opioid use disorders thus might not be as high as they appeared *a priori*.

## Conclusions

Congressional recognition of the need for pharmacotherapeutics for prevention and treatment of substance use disorders and the difficulties of such development led to establishment of the medications development program at the National Institute on Drug Abuse in 1990, and to its increasing funding and sophistication during subsequent years.^13^ Bases for regulation of substances with abuse liability dates to the early 1900's and currently centers on the Controlled Substance Act and related legislation.^14^ Despite this regulatory sophistication and support for medication development, there is still no licensed pharmacotherapeutic that can prevent development of substance use disorders in those who are prescribed stimulants or opioids and none that can effectively treat established stimulant use disorders.

In these contexts, development of pentilludin, a novel pharmacotherapeutic that acts at a novel addiction-associated site to reduce reward from stimulants and opioids, provides an example of the promises and hurdles that face antiaddiction medication development. Categorizing pentilludin's strengths and limitations in several potential settings and with several different sets of endpoints outlines some of the ways in which choosing the correct set of hurdles and endpoints could increase the likelihood that this compound will be able to reach general clinical use.

## Author contributions

The author confirms being the sole contributor of this work and has approved it for publication.

## Footnotes

^1^ https://clincalc.com/drugstats/drugs/amphetamine

^2^ https://www.samhas.gov/data/data-we-collect/teds-treatment-episode-data-set

^3^ https://www.cdc.gov/drugoverdose/rxrate-maps/index.html

^4^https://www.hhs.gov/opioids/statistics/index.html; https://nida.nih.gov/research-topics/opioids

^5^ https://samhsa.gov/data/release/2020-national-survey-drug-use-health-nsduh-release

^6^ https://samhsa.gov/data/data-we-collect/n-ssats-national-survey-substance-abuse-treatment-services

^7^ https://mouse.brain-map.org

^8^ https://www.samhas.gov/data/data-we-collect/teds-treatment-episode-data-set

^9^ https://www.fda.gov/industry/developing-products-rare-diseases-conditions/designating-orphan-product-drugs-and-biological-products

^10^ https://www.dea.gov/drug-information/drug-scheduling

^11^ https://www.fda.gov/media/116739/download

^12^ https://nida.nih.gov/publications/drugs-brains-behavior-science-addiction/drug-misuse-addiction

^13^ https://nida.nih.gov/about-nida/organization/divisions/division-therapetics-medical-consesquences-dtmc/research-programs

^14^ https://uscode.house.gov/view.xhtml?path=/prelim@title21/chapter13&edition=prelim
